# Evolution of idiopathic membranous nephropathy to class v lupus nephritis: a case report and literature review

**DOI:** 10.3389/fmed.2026.1801837

**Published:** 2026-03-23

**Authors:** Bo Shen, Cheng Peng, Weicheng Xiao

**Affiliations:** 1 Department of Blood Purification Center, Shaoxing Second Hospital, Shaoxing, Zhejiang, China; 2 Department of Nephrology, Shaoxing Second Hospital, Shaoxing, Zhejiang, China

**Keywords:** anti-PLA2R antibodies, case report, class V lupus nephritis, idiopathic membranous nephropathy, obinutuzumab

## Abstract

**Background:**

The M-type phospholipase A2 receptor (PLA2R) antibody positivity is considered a specific serological marker for idiopathic membranous nephropathy (IMN), and its presence in class V lupus nephritis (LN) is rare. There is limited reporting on the longitudinal progression from IMN to systemic lupus erythematosus (SLE) with class V LN, particularly in cases with persistently positive anti-PLA2R antibodies.

**Case presentation:**

A 54-year-old woman presented with nephrotic syndrome in 2016, with initial renal biopsy showing podocyte disease. In 2020, disease recurrence with positive serum anti-PLA2R antibodies led to a second biopsy diagnosing IMN stage II. In 2024, she presented with edema and facial erythema. Serology revealed new-onset positive ANA and anti-dsDNA antibodies alongside persistent anti-PLA2R positivity, meeting SLE criteria. A third renal biopsy confirmed class V LN. After treatment with methylprednisolone and hydroxychloroquine, and following a rituximab infusion reaction, she achieved clinical remission with obinutuzumab.

**Conclusion:**

This case highlights that positive anti-PLA2R antibodies do not definitively exclude secondary MN such as class V LN. Long-term serological monitoring and repeat renal biopsy should be considered in IMN patients with atypical clinical evolution to identify potential transformation to LN.

## Introduction

Membranous nephropathy (MN) is a type of glomerular disease characterized by immune complex deposition beneath the glomerular epithelium and diffuse thickening of the basement membrane (1). Clinically, it often presents as nephrotic syndrome. In 2009, the M-type phospholipase A2 receptor (PLA2R) was first identified as the target antigen for idiopathic MN (IMN) (2). To date, more than 10 types of MN target antigens have been discovered (3). Numerous studies have confirmed that serum anti-PLA2R antibodies and glomerular PLA2R expression are not only the most important diagnostic markers for IMN, but also closely related to disease activity, progression risk, and long-term outcomes (4). However, subsequent studies have also found that a small number of secondary MN patients also have positive serum anti-PLA2R antibodies (5, 6).

Class V lupus nephritis (LN) is the most common secondary membranous nephritis (SMN) (7), with an incidence rate of approximately 10 to 20% among LN cases (8). The reported target antigens include extracellular matrix protein 1/2 (EXT) 1/2, neural cell adhesion molecule 1 (NCAM1), etc (3). The expression of different antigens in renal tissues of class V LN is associated with the heterogeneity of clinical pathological features and prognosis. For example, the urine protein level of patients with glomerular EXT1/2 positive class V LN is significantly higher than that of EXT-negative class V LN, and the long-term prognosis is better; NCAM1 is related to the central nervous system damage of patients with class V LN and class V + III/IV LN (9, 10).

Previous studies have shown that in patients with V-type LN, the serum anti-PLA2R antibodies and the PLA2R staining in renal tissues are usually negative (11, 12). However, recent literature reports that a few patients with V-type LN may also have positive serum anti-PLA2R antibodies or positive PLA2R expression in renal tissues (13). To further explore this special clinical and pathological manifestation, this article reports a case of membranous LN that lasted for 8 years, progressing from minimal change disease (MCD) to IMN and eventually turning positive for serum anti-PLA2R antibodies. It also combines relevant literature to review the clinical characteristics and treatment strategies of this case, in order to enhance the understanding of the transformation from IMN to LN.

## Case presentation

**Figure 1:**
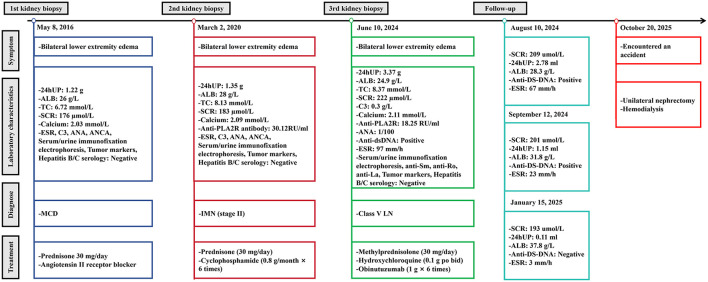
The timeline for diagnosis and treatment

**Figure 2:**
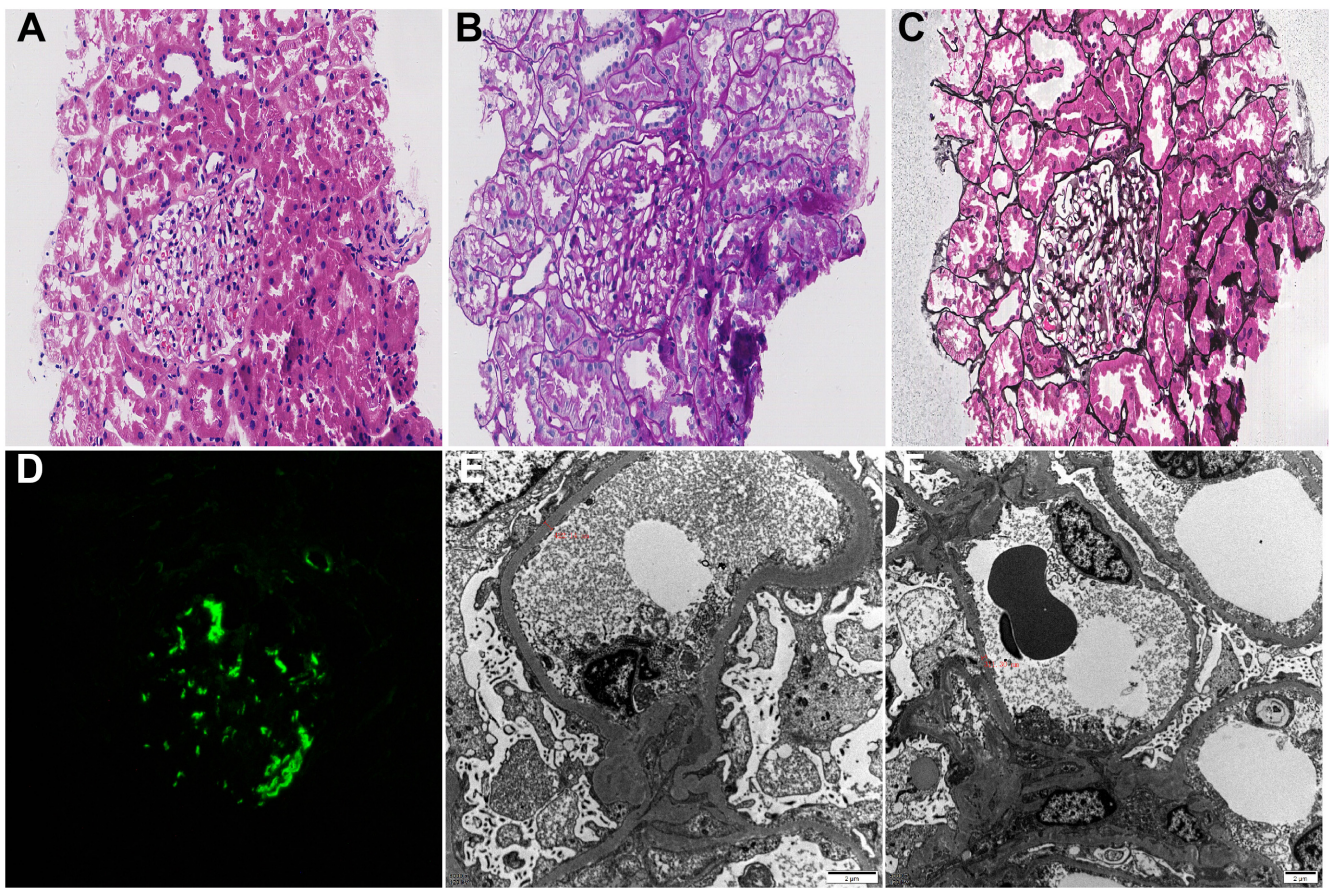
Biopsy findings (2016). **(A)** Hematoxylin-eosin staining (× 200): vacuolar degeneration of renal tubular epithelial cells with interstitial inflammatory cell infiltration; **(B)** Periodic acid schiff staining (× 400): mild mesangial proliferation and renal tubular dilation; **(C)** Periodic acid silver methenamine staining (× 400): no obvious glomerular hemosiderin deposition; **(D)** Immunofluorescence staining for C1q (× 200); **(E)** Electron microscopy (× 8,000):diffuse podocyte foot process fusion with segmental glomerular basement membrane thickening, bar = 2 μm; **(F)** Electron microscopy (× 5,000): podocyte vacuolar degeneration with sparse mesangial electron-dense deposits, bar = 2μm.

On May 8, 2016, a 54-year-old woman was admitted to the Nephrology Department of Shaoxing Second Hospital for 1 week of bilateral lower extremity edema. Physical examination revealed blood pressure of 138/84 mmHg and mild lower extremity edema. Laboratory findings included: 24-h urine protein (24 h UP) 1.22 g, albumin (ALB) 26 g/L, total cholesterol (TC) 6.72 mmol/L, and serum creatinine (Scr) 176 μmol/L. Tumor markers, antinuclear antibody (ANA), ANCA, serum immunofixation electrophoresis, and hepatitis B/C serology were all negative. Other parameters are shown in Figure 1. On May 11, renal biopsy showed 19 glomeruli without global or segmental sclerosis. PASM and Masson stains showed no definite subepithelial electron-dense deposits (i.e., no immune complex deposition). Tubular epithelial cells exhibited vacuolar and granular degeneration. Immunofluorescence revealed weak IgG and C1q staining (±). Electron microscopy demonstrated segmental basement membrane thickening (up to 550 nm), mesangial hypercellularity and matrix expansion, and diffuse podocyte foot process effacement (>80%) with microvillous transformation and vacuolization. The diagnosis was MCD (Figure 2). Treatment with prednisone 30 mg/day plus an angiotensin II receptor blocker led to complete remission of proteinuria within 4 months, sustained for 4 years.

**Figure 3:**
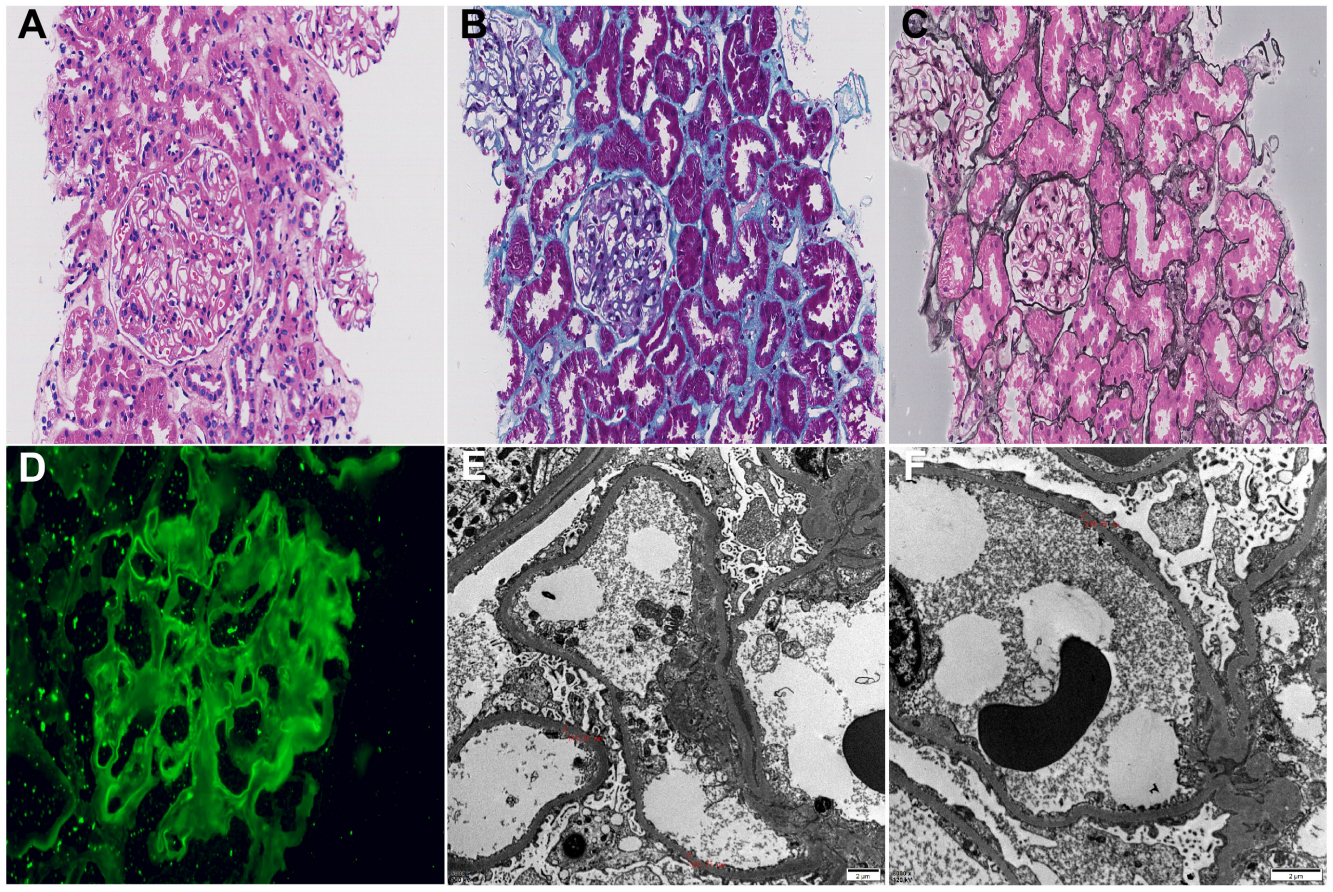
Biopsy findings (2020). **(A)** Hematoxylin-eosin staining (× 200): no glomerulosclerosis; tubular epithelial degeneration with interstitial inflammatory cell infiltration; **(B)** Masson's trichrome staining (× 400): subepithelial red-brown deposits in the glomerular basement membrane; **(C)** Periodic acid silver methenamine staining (× 400): Epithelial-side spike formation in the glomerular basement membrane; **(D)** Immunofluorescence staining for PLA2R (× 400); **(E)** Electron microscopy (× 5,000): subepithelial electron-dense deposits with foot process fusion, bar = 2 μm; **(F)** Electron microscopy (× 8,000): basement membrane thickening with subepithelial electron-dense deposits and spikes, bar = 2 μm.

On March 2, 2020, the patient was readmitted with bilateral lower limb edema. The laboratory tests were as follows: 24 h UP was 1.35 g, ALB was 28 g/L, TC was 8.13 mmol/L, Scr was 183 μmol/L, and anti-PLA2R antibody was 30.12 RU/ml. Other laboratory parameters are shown in Figure 1. Given the seropositivity and discordance with the previous pathological diagnosis 4 years earlier, a repeat renal biopsy was performed on March 6, 2020. Light microscopy of 15 glomeruli revealed no sclerosis; Masson staining showed subepithelial eosinophilic deposits, and PASM staining exhibited basement membrane ‘spikes'. Immunofluorescence revealed IgG (2 +), C3 (2 +), IgG1 (3 +), IgG4 (2 +), PLA2R (2 +) and C1q (–). Electron microscopy confirmed irregular glomerular basement membrane thickening (240nm−700 nm) with extensive subepithelial and intramembranous electron-dense deposits, consistent with IMN stage II (Figure 3). The patient was treated with prednisone (30 mg/day) and cyclophosphamide (0.8 g/month × 6 times). Proteinuria gradually resolved within 6 months and remained negative over 4 years of follow-up.

**Figure 4:**
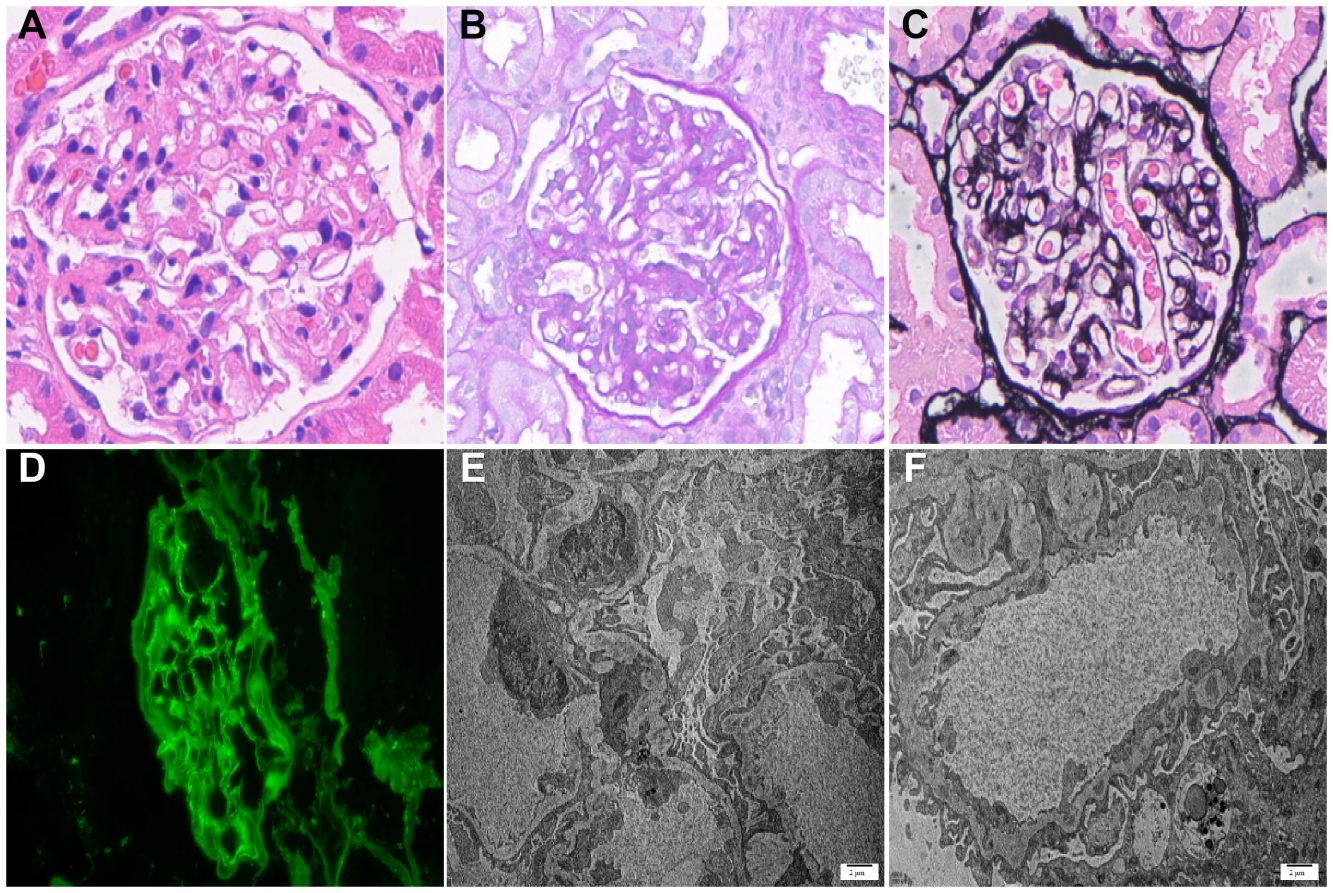
Biopsy findings. **(A)** Hematoxylin-eosin staining (× 400): mild mesangial cell proliferation and matrix expansion with capillary loop infiltration; **(B)** Periodic acid schiff staining (× 400): glomerular capillary basement membrane thickening and mesangial matrix expansion; **(C)** Periodic acid silver methenamine staining (× 400): glomerular basement membrane spikes with mesangial hypercellularity and matrix expansion; **(D)** Immunofluorescence staining for PLA2R (× 400); **(E)** Electron microscopy (× 6,000): moderate mesangial electron-dense deposits with podocyte foot process effacement, bar = 2μm; **(F)** Electron microscopy (× 8,000): subepithelial and intramembranous electron-dense deposits with podocyte cytoplasmic vacuolization, bar = 2μm.

On June 10, 2024, the patient presented with bilateral lower extremity edema and facial erythema lasting 3 days. Physical examination confirmed facial erythema and moderate bilateral edema. Laboratory tests showed a 24 h UP of 3.37 g, ALB of 24.9 g/L, TC of 8.37 mmol/L, calcium of 2.11 mmol/L, Scr of 222 μmol/L, C3 of 0.3 g/L, and ESR of 97 mm/h. ANA was positive at 1/100, presenting as homogeneous type. Anti-dsDNA antibody was positive, and anti-PLA2R antibody was 18.25 RU/ml. Other laboratory parameters are shown in Figure 1. Based on the 2019 European League Against Rheumatism/American College of Rheumatology classification criteria for SLE(14), the patient met the ANA positivity entry criterion (≥1:80) and scored 23 points total: facial erythema (acute cutaneous lupus, 6 points), biopsy-proven class V lupus nephritis (8 points), anti-dsDNA antibodies (6 points), and low C3 (3 points). With a score ≥10, SLE was classified. A third renal biopsy was performed on June 16 to confirm LN and guide treatment. Light microscopy of 18 glomeruli showed no sclerosis, with mild mesangial cell and matrix proliferation, thickened and rigid capillary basement membranes, and small vacuoles with spiculated protrusions. Immunofluorescence revealed IgG (2 +), IgG1 (2 +), IgG2 (±), IgG4 (2 +), IgA (±), IgM ( +), C1q (1–2 +), C3 (2 +), Kappa (2 +), Lambda (2 +), PLA2R (–), THSD7A (–), ETX1 (–), and ETX2 (–). Electron microscopy confirmed electron-dense deposits in the mesangial, subepithelial, intramembranous, and endothelial regions, accompanied by extensive podocyte foot process effacement. Integrated with clinical findings, the pathological diagnosis was class V LN (Figure 4). Treatment included methylprednisolone (30 mg/day), hydroxychloroquine (0.1 g po bid), and planned rituximab (RTX). However, during the first RTX infusion (1 g) on June 22, the patient developed chills and fever, leading to its discontinuation due to a suspected infusion reaction. The patient subsequently received obinutuzumab (1 g) on June 24 and July 9. Follow-up showed progressive reduction in 24 h UP: 2.78 g (August 10), 1.15 g (September 12), and 0.11 g (January 15, 2025), with ALB rising to 37.8 g/L. These findings indicate successful remission of LN. In October 2025, the patient sustained severe impact and compression in a severe motor vehicle accident, causing direct rupture of one kidney's parenchyma and requiring a unilateral nephrectomy. A delayed subcapsular hematoma developed in the right kidney 48 h post-accident, causing renal compression. Given the patient's poor overall condition, which ruled out another surgery in the short term, the family chose conservative management. Antibacterial and analgesic medications used during treatment directly damaged the renal tubules, and this, combined with the initial trauma, accelerated the progression to renal failure. Subsequently, due to trauma-related renal loss and increased load on the remaining kidney, Scr levels rose and stabilized between 550–700 μmol/L, consistent with end-stage kidney disease (ESKD). Given the irreversible loss of renal function and persistent uremic symptoms, the patient initiated maintenance hemodialysis. The diagnostic and treatment timeline is summarized in Figure 1.

## Discussion and conclusion

IMN is a type of glomerular disease characterized by the deposition of endogenous immune complexes in the glomerular basement membrane. It accounts for 70%−80% of MN (15) and 20%−30% of primary glomerular diseases (16). SMN is induced by specific causes such as autoimmune diseases, malignant tumors, infections, and drug exposure, accounting for 20%−30% of MN (17). LN, as an important type of SMN, accounts for 10%−15% of SMN (18), and its pathological features include multiple sites of immune complex deposition (mesangial area, subepithelial area, and subendothelial area), accompanied by complement activation and serological abnormalities such as ANA, anti-dsDNA antibodies, etc. (19, 20), which have essential differences in immune mechanism and pathological phenotype from IMN. Currently, there have been a few case reports indicating that IMN can gradually progress to LN. This article reports a case where foot cell lesion progressed to IMN and eventually transformed into class V LN.

This case presents a typical “three-step” pathological evolution trajectory: The first biopsy in 2016 indicated MCD. In 2020, due to recurrence of proteinuria, combined with positive anti-PLA2R antibodies and renal biopsy, it was diagnosed as IMN stage II. In 2024, systemic manifestations of SLE (facial erythema) appeared, and anti-PLA2R remained positive. New serological abnormalities (positive ANA, anti-dsDNA antibodies, and decreased complement C3) occurred. The third biopsy confirmed class V LN. This disease course has the following three characteristics: firstly, the total duration of the disease is 9 years, with two clinical remission periods lasting 4 years, respectively. The conversion interval is longer than the average 6.92 ± 5.32 years reported in the literature (21–25); secondly, the initial manifestation was isolated MCD, gradually progressing to immune complex-mediated glomerular diseases, suggesting that immune abnormalities are gradually expanding; thirdly, after the patient was intolerant to RTX, they switched to obinutuzumab and achieved complete clinical remission, providing a reference for the treatment of class V LN.

As of January 2026, a total of 12 cases of IMN progressing to LN were included in PubMed (Tables 1–2). The majority of these cases were reported from China (7 cases) (21, 22), followed by the United States (4 cases) (24, 25) and Turkey (1 case) (23); among them, 11 were female and 1 was male. The average age at the time of transformation was 38.7 years, with a disease duration ranging from 2 to 16 years. In terms of laboratory indicators, 24 h UP of patients in the initial stage of IMN was mostly 2.2–8.67 g, and SCR was mostly normal or slightly elevated; when transforming to LN, 24 h UP fluctuated between 0.48–8.1 g, and only 1 case showed a significant increase in SCR (up to 663 μmol/L), which might be related to lupus activity. After emergency hemodialysis, renal function partially recovered. The common characteristics of this case and the literature reports include: most patients had no SLE-related serological abnormalities in the initial stage, and all showed positive ANA at the time of transformation, among which 8 cases were clearly recorded as positive anti-dsDNA antibodies; in terms of immunofluorescence, the initial stage was mainly characterized by IgG and C3 deposition, with some accompanied by IgG1, IgG4, and C1q deposition. After transformation, multiple antibody complexes were deposited (based on IgG, C3, and C1q, with some combined with IgA, IgM, and light chain deposition), and 2 cases detected PLA2R antigen deposition. The pathological transformation was mainly pure membranous (V type) or mixed type (III+V/IV+V/III-V) LN, accounting for approximately 50%. In terms of treatment plans, 11 patients with clear records mostly used traditional immunosuppressants such as glucocorticoids combined with cyclophosphamide, mycophenolate mofetil, tacrolimus or leflunomide. Due to an allergy to RTX, this case was switched to obinutuzumab targeted therapy. After treatment, 24 h UP decreased from 3.37 to 0.11 g, and SCR remained stable, achieving complete remission. A randomized controlled trial involving 271 patients with LN showed that obinutuzumab combined with standard treatment was superior to standard treatment alone in reducing proteinuria and protecting renal function (26). A recent Meta-analysis also suggested that obinutuzumab combined with standard treatment might be superior to existing standard regimens in terms of efficacy and safety (27). This case further verified the potential efficacy of this drug in class V LN. Compared with the 12 previously reported cases of IMN progressing to LN, the present case is distinctive in three aspects: it originated from MCD rather than IMN, maintained anti-PLA2R seropositivity through the diagnosis of class V LN, and employed obinutuzumab as a contemporary B-cell-targeting therapy instead of traditional immunosuppression.

**Table 1:** Summary of clinical data of cases where IMN progressed to LN.

**References**	**Biopsy no**.	**Gender**	**Country**	**Age (year)**	**24 h UP (g)**	**Scr (μmol/L)**	**PLA2R Ab (RU/ml)**	**ANA**	**Anti-dsDNA (IU/ml)**
Zhang et al.(21)	1st	F	China	34	3.8	135	/	–	–
	2nd			43	8.1	81	/	–	–
	3rd			50	1.5	663	1.64	1/80	>200
Jin et al. case 1(22)	1st	F	China	25	4.37	45	/	1/80	–
	2nd			32	2.76	46	–	1/640	55.2
Jin et al. case 2(22)	1st	F	China	34	7	/	/	/	/
	2nd			46	3.0.59	62	2.05	1/640	–
Jin et al. case 3(22)	1st	F	China	45	8.67	50	135.5	1/80	–
	2nd			46	2.4	60	/	1/640	73.1
Jin et al. case 4(22)	1st	F	China	21	5.75	50	–	1/80	–
	2nd			23	4.57	39	1.8	1/320	32.7
Jin et al. case 5(22)	1st	F	China	37	2.98	50	/	1/80	–
	2nd			51	5.65	52	1.8	1/640	0.81
Jin et al. case 6(22)	1st	M	China	58	5.73	58	/	–	–
	2nd			74	0.48	74	0.47	1/320	47.8
Gokalp et al.(23)	1st	M	Turkey	47	5.02	N	–	–	–
	2nd			59	4.4	/	27.12	+	0.73
Kallen et al.case 1(24)	1st	F	US	7	2.2	N	/	1/100	0.47
	2nd			8	/	N	/	1/200	–
Kallen et al.case 2(24)	1st	F	US	14	/	/	/	–	–
	2nd			17	/	/	/	1/800	/
Shearn et al.case 1(25)	1st	F	US	25	3.2	/	/	–	–
	2nd			29	/	/	/	1/160	/
Shearn et al.case 2(25)	1st	F	US	25	4.8	/	/	–	–
	2nd			29	/	/	/	1/160	–

US, United States; 24 h UP, 24-h urine protein; Scr, serum creatinine; Anti-PLA2R, anti-phospholipase A2 receptor antibody; ANA, antinuclear antibody; anti-dsDNA, anti-double-stranded DNA antibody; F, female; M, male;/, unknown; –, negative; +, positive.

**Table 2:** Summary of pathology and treatment options of cases where IMN progressed to LN.

**References**	**Biopsy no**.	**Pathological diagnosis**	**Immunofluorescence**	**Treatment option**				
				**PRED**	**CYC**	**TAC**	**LEF**	**MMF**
Zhang et al.(21)	1st	MCD	/	A	A			
	2nd	IMN (II)	IgG, C3, IgG1, IgG4, C1q, λ, k	A	A			
	3rd	LN (IV+V)	IgG, C3, C1q, IgG1, IgA, IgM, IgG2, IgG3, λ, k	A	A			
Jin et al. case 1(22)	1st	IMN (I-II)	IgG, C3, C1q	AB	A		AB	
	2nd	LN III+IV)	IgG, C3, C1q, IgA, IgM, IgG2, λ, k	A		A		A
Jin et al. case 2(22)	1st	IMN	/	AB	A	B		
	2nd	LN (V)	IgG, C3, C1q, IgG1, IgG2, IgG3, IgG4, IgA, IgM, λ, k, PLA2R	A		A		A
Jin et al. case 3(22)	1st	IMN (I-II)	IgG, C3, C1q, IgG1, IgA, IgM, λ, k, PLA2R	A			A	
	2nd	LN	/	A				A
Jin et al. case 4(22)	1st	IMN (I-II)	IgG, C3, C1q, IgG1, IgG2, IgG4, IgM, λ, k			A		
	2nd	LN (V)	IgG, C3, C1q, IgG1, IgG2, IgG3, IgG4, IgA, IgM, λ, k	A				A
Jin et al. case 5(22)	1st	IMN	IgG, C3, IgM	AB	A		A	
	2nd	LN (III-V)	IgG, C3, C1q, IgG1, IgG2, IgG3, IgG4, IgA, IgM, λ, k	A			A	
Jin et al. case 6(22)	1st	IMN (I-II)	IgG, C3, IgM	A		A		
	2nd	LN	/			A		
Gokalp et al.(23)	1st	IMN	IgG, C3	/	/	/	/	/
	2nd	LN (V)	IgG, C3, C1q, IgA, IgM, λ, k	/	/	/	/	/
Kallen et al.case 1(24)	1st	IMN	IgG, C3	/	/	/	/	/
	2nd	LN	IgG, C3, IgA, IgM, Fibrin	A	A			
Kallen et al.case 2(24)	1st	IMN	Typical change	/	/	/	/	/
	2nd	LN	IgG, C4, IgA	A				
Shearn et al.case 1(25)	1st	IMN	/	/	/	/	/	/
	2nd	LN	IgG, C3	/	/	/	/	/
Shearn et al.case 2(25)	1st	IMN	IgG, C3, C4, IgA, IgM	/	/	/	/	/
	2nd	LN	/	/	/	/	/	/

MCD, minimal change disease; IMN, idiopathic membranous nephropathy; LN, lupus nephritis; PRED, prednisone; CYC, cyclophosphamide; TAC, tacrolimus; LEF, leflunomide; MMF, mycophenolate mofetil; F, female; M, male;/, unknown; A, first-line treatment; B, second-line treatment.

In addition, the initial manifestation of this case was MCD, which is relatively rare among similar transformed cases. Only Zhang et al. reported a case where minimal change nephropathy progressed to IMN and then transformed into LN (21), suggesting that the immune phenotype of glomerular diseases may have a dynamic evolution spectrum. The evolution trajectory of “MCD—IMN—LN” in this case may reflect a progressive expansion of the autoimmune response. In the initial stage of MCD, immunofluorescence showed only weak positive IgG and C1q, and electron microscopy suggested extensive fusion of foot processes, without clear immune complex deposition. In 2020, when it progressed to IMN, anti-PLA2R antibodies were positive and subepithelial immune complex deposition occurred, suggesting that B cell activation produced specific antibodies, initiating an autoimmune response against podocytes (28). In 2024, when it progressed to LN, the third biopsy showed multiple sites of immune complex deposition, indicating a transition from a localized podocyte-directed immune response to a systemic autoimmune disorder (29). Moreover, the PLA2R deposition in the renal tissue turned negative, but the serum anti-PLA2R antibody persisted after the transformation. The persistence of serum anti-PLA2R antibodies at class V LN diagnosis (18.25 RU/ml) raises questions about their role in evolving SLE. Immune cross-reactivity with lupus autoantigens cannot be ruled out but remains speculative without direct experimental support (30). Alternatively, the dissociation between persistent serum antibodies and negative glomerular PLA2R staining on the third biopsy suggests that in the class V LN phase, the antibody may no longer target its renal antigen (4, 31). Due to the retrospective nature, anti-PLA2R titers were not rechecked at clinical remission in January 2025, a limitation that prevents assessment of whether the antibody tracks with disease activity or represents an epiphenomenon. Serial anti-PLA2R measurements in future prospective studies of IMN-to-LN transition cases would help clarify its trajectory and pathogenic role. In addition, Qin et al. reported that in 20 cases of class V LN patients, 1 case had a positive serum anti-PLA2R antibody (32); Garcia-Vives et al. also found that in 190 cases of systemic lupus erythematosus patients, 10 cases had a positive serum anti-PLA2R antibody, among which 7 cases were V-type LN(13). These observations suggest that positive anti-PLA2R antibody cannot be an absolute basis for excluding LN. Similarly, Sama et al. reported PLA2R-negative membranous nephropathy as a rare renal manifestation of IgG4-related disease, a condition that more typically presents with IgG4-rich tubulointerstitial nephritis (33). This example underscores that both PLA2R positivity and negativity must be interpreted in the broader clinical and serologic context when distinguishing primary from secondary membranous nephropathy.

Although sequential biopsies and clinical course support true transformation from IMN to class V LN, alternative explanations remain possible. First, subclinical SLE may have been present from the outset, with initial MCD and later IMN representing early, organ-limited manifestations of an underlying autoimmune diathesis that subsequently evolved into overt SLE, consistent with known delays between autoantibody emergence (e.g., ANA, anti-dsDNA) and clinical diagnosis. Second, coexistence of IMN and class V LN cannot be ruled out: the patient may have developed anti-PLA2R–positive IMN and later independently developed SLE with class V lupus nephritis; persistent anti-PLA2R antibodies could then reflect a coincidental, non-pathogenic response unrelated to lupus activity. Third, anti-PLA2R antibodies are occasionally detected in LN, as demonstrated by Garcia-Vives et al. and others, meaning PLA2R positivity does not exclude lupus-associated MN. In this case, loss of glomerular PLA2R staining on the third biopsy despite persistent serum antibodies adds further complexity. Without prospective pre-onset immunological monitoring or specific biomarkers to distinguish these entities, no single explanation can be definitively confirmed. However, the prolonged latency between renal presentations, progression from isolated podocyte injury to systemic autoimmunity, and supporting literature cases (Tables 1–2) collectively favor an evolving autoimmune process over simple coexistence.

Based on the analysis of this case and the literature, the following clinical implications are proposed: Firstly, for patients with an initial diagnosis of IMN, even if anti-PLA2R antibodies are positive and there are no manifestations of SLE, long-term follow-up of serological indicators (ANA, anti-dsDNA antibodies, complement) and changes in proteinuria is still necessary. The 2024 LN guidelines of Kidney Disease: Improving Global Outcomes (KDIGO) also emphasize that for patients suspected of having secondary glomerular diseases, the autoantibody profile should be dynamically monitored to avoid missed diagnosis of delayed autoimmune diseases (34). Secondly, when IMN patients experience proteinuria recurrence, especially accompanied by unexplained rashes and decreased complement levels, timely renal biopsy should be performed for re-evaluation to determine if the pathological type has transformed, to avoid disease progression due to inappropriate treatment plans. Thirdly, for LN patients who experience infusion reactions after using RTX, obinutuzumab can be used as an alternative treatment option, but more large-sample studies are needed to verify its long-term efficacy and safety.

In conclusion, this article reports a case with a disease course of 8 years, starting from MCD and gradually progressing to IMN, and eventually transforming into class V LN. We believe that even if the anti-PLA2R antibody test is positive, the diagnosis of IMN should not rule out the possibility of SMN (such as LN), and the autoantibody profile should be dynamically monitored. In necessary cases, renal biopsy should be performed.

## Data Availability

The original contributions presented in the study are included in the article/supplementary material, further inquiries can be directed to the corresponding author.
